# Aortic remodelling based on false lumen communications in patients undergoing acute type I dissection repair with AMDS hybrid prosthesis: a substudy of the DARTS trial

**DOI:** 10.1093/ejcts/ezae194

**Published:** 2024-05-17

**Authors:** Ryaan EL-Andari, Sabin J Bozso, Jeevan Nagendran, Jennifer Chung, Maral Ouzounian, Michael C Moon

**Affiliations:** Division of Cardiac Surgery, Department of Surgery, University of Alberta, Edmonton, AB, Canada; Division of Cardiac Surgery, Department of Surgery, University of Alberta, Edmonton, AB, Canada; Division of Cardiac Surgery, Department of Surgery, University of Alberta, Edmonton, AB, Canada; Division of Cardiac Surgery, University of Toronto, Toronto, ON, Canada; Division of Cardiac Surgery, University of Toronto, Toronto, ON, Canada; Division of Cardiac Surgery, Department of Surgery, University of Alberta, Edmonton, AB, Canada

**Keywords:** Aorta, Aortic remodelling, Aortic dissection, AMDS

## Abstract

**OBJECTIVES:**

The Dissected Aorta Repair Through Stent (DARTS) Implantation trial demonstrated positive proximal aortic remodelling following aortic dissection repair with the AMDS hybrid prosthesis. In this study, we look to identify predictors of aortic remodelling following aortic dissection repair with AMDS including whether communications between branch vessels and the false lumen (FL) predict aortic growth.

**METHODS:**

The DARTS implantation trial included patients who underwent acute DeBakey type I aortic dissection (ATAD I) repair with the AMDS from March 2017 to January 2019. Anatomic measurements were collected from original computerized tomography scans. Measurements were taken at zones 2, 3, 6 and 9. Patients were grouped based on the number of FL communications with the supra-aortic branch vessels or visceral branch vessels.

**RESULTS:**

Forty-seven patients were included in the original DARTS implantation trial. Patients with FL communications with the supra-aortic branch vessels tended to have significant growth at zone 3 (*P* = 0.02–0.0018), while greater numbers of visceral FL communications tended to predict aortic growth at zones 3 (*P* = 0.003), 6 (*P* = 0.017–0.0087) and 9 (*P* = 0.0016–0.0003).

**CONCLUSIONS:**

Aortic remodelling following ATAD I repair using the AMDS may be predicted by local FL communications with branch vessels. Patients undergoing ATAD I repair were more likely to experience significant aortic growth in zone 3 with more head vessel communications and in zones 3, 6 and 9 with more visceral FL communications. Predictors of aortic remodelling may help to guide initial surgical management for aortic dissection patients.

## Graphical Abstract

**Figure:**
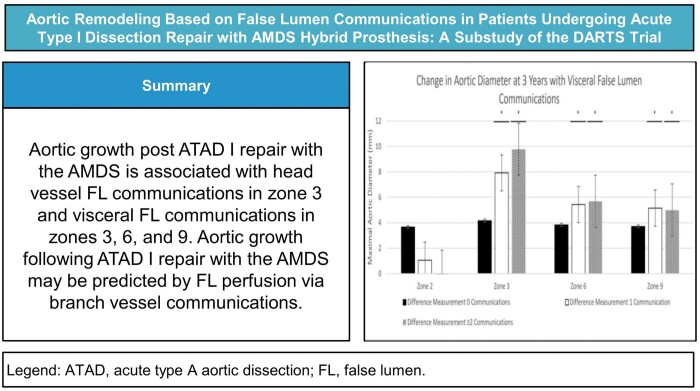

## INTRODUCTION

Acute DeBakey type I aortic dissection (ATAD I) is a life-threatening condition requiring emergent surgical intervention to prevent loss of life [1, 2]. Several surgical approaches exist in the management of ATAD I. The ascending hemiarch repair with resection of the entry tear is the standard life-saving intervention for ATAD I [1, 2]. Extended arch repairs such as the total arch repair, zone 2 debranching with antegrade thoracic endovascular aortic repair and various other hybrid techniques are used in patients with distal intimal tears, more extensive disease or high-risk patients such as those with connective tissue disease [1–8]. Extended arch repairs require longer operative times and may increase the risk of postoperative complications.

Novel technologies in the form of the AMDS hybrid prosthesis have been developed in recent years to aid in the treatment of patients with ATAD I. The Dissected Aorta Repair Through Stent (DARTS) implantation demonstrated the safety and efficacy of the AMDS hybrid prosthesis with positive aortic remodelling proximally [9–11]. Distal aortic remodelling, while also favourable, was less consistent.

While previous literature has identified anatomic predictors of aortic growth following aortic dissection repair such as patent or partially thrombosed FL, multiple entry tears, FL ejection fraction, as well as size and shape of the dissected aorta [4, 12–16], the vast majority of these studies have focused on type B aortic dissection and it remains unclear which anatomic features at the time of ATAD I predict proximal and distal growth post dissection repair. Furthermore, while the original DARTS trial identified positive aortic remodelling with partial or total proximal FL thrombosis, predictors for aortic remodelling following ATAD I repair with the AMDS have not been identified. The objective of this study is to elucidate how aortic remodelling is impacted by preoperative FL communications, in the aorta and branch vessels, following ATAD I repair with the AMDS.

## METHODS

The DARTS implantation trial is a multicentre, prospective, nonrandomized, single-arm trial demonstrating the safety and efficacy of the AMDS allowing for effective sealing of the distal anastomosis and resulted in positive remodelling of the aortic arch in patients with ATAD I. The methods and results of this trial have previously been described [9–11]. The AMDS hybrid prosthesis (Artivion, Georgia, USA) is a hybrid stent graft designed to induce positive aortic remodelling in patients undergoing repair of ATAD I [9–11]. The AMDS provides benefits to aortic remodelling without significantly increasing the complexity or length of the standard hemiarch repair [9–11]. The majority of patients had reduced or stable total aortic diameter and false lumen (FL) partial or complete thrombosis in zones 0–3 [9–11].

### Imaging

Preoperative and postoperative computerized tomography (CT) scans were performed by each participating institution at each prespecified follow-up period. The CT scans were then sent to an independent Core Lab that retrospectively reviewed and evaluated TeraRecon analysis software (TeraRecon, Durham, NC). Data collected from the CT scans included the location of entry tears, aortic anatomy, location of true lumen to FL communications, FL patency and maximal aortic diameter at various locations (Table 1). Postoperative measurements were taken from the trial data at 3 years of follow-up to be compared to the preoperative imaging. The clinical data utilized in this study were collected from the original dataset including maximal aortic diameter at various sites in the aorta as well as the number of communications between the FL with the head vessels (i.e. innominate artery, left common carotid artery, left subclavian artery) or visceral branch vessels (i.e. coeliac artery, superior mesenteric artery, right renal artery, left renal artery).

**Table 1:** Aortic true and false lumen status at 3 years of follow-up

Aortic zone	Complete false lumen thrombosis	Partial false lumen thrombosis	Patent false lumen
0	11/26 (42.3%)	13/26 (50.0%)	2/26 (7.7%)
1	8/25 (32.0%)	6/25 (24.0%)	11/25 (44.0%)
2	9/27 (33.3%)	8/27 (29.6%)	10/27 (37.0%)
3	6/28 (21.4%)	13/28 (46.4%)	9/28 (32.1%)
4	3/25 (12.0%)	19/25 (76.0%)	3/25 (12.0%)
5	2/23 (8.7%)	19/23 (82.6%)	2/23 (8.7%)

### Definitions

Entry tears are defined as the initial intimal tears in the intima of the aorta leading to infiltration of the media by blood and aortic dissection [17]. Re-entry tears are defined as subsequent distal tears in the aorta relieving pressure in the FL and allowing for flow from the higher pressure FL into the lower pressure true lumen. Distal anastomotic new entry tears are defined as entry tears arising at the distal aortic anastomosis on postoperative imaging.

### Analysis

**Figure 1:**
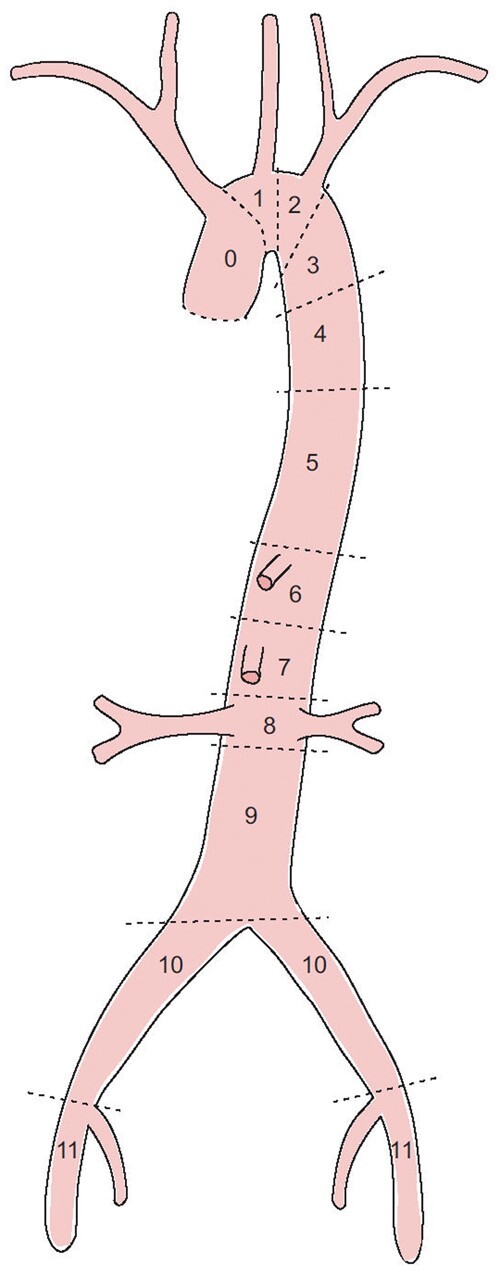
Illustration of the aorta divided into zones.

**Figure 2:**
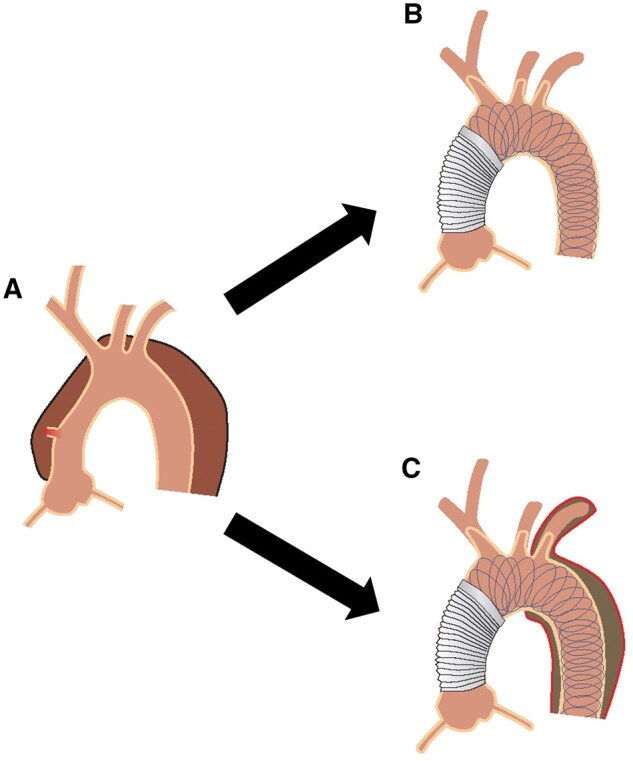
Illustration of an aortic dissection (**A**), a successful postoperative repair with the AMDS hybrid prosthesis (**B**) and a postoperative repair with the AMDS hybrid prosthesis with residual false lumen and adverse remodelling (**C**).

All 29 patients with 3-year CT scans had CT measurements available for the majority of locations. In cases where data were missing for specific measurements, both pre- and postoperative measurements were removed for that patient at that location. Patients were grouped based on the number of branch vessel communications with the FL at various sites in the aorta including those with 0, 1 or ≥2 branch vessel communications. Measures of aortic growth utilizing changes in aortic diameter at zones 2, 3, 6 and 9 were compared between groups (Figs 1 and 2). One factor analysis of variance (ANOVA) tests were performed comparing the absolute changes in aortic diameter at each location. All groups were assessed for normality between comparisons using a Jarque–Bera test with a *P*-value of <0.05 indicating non-normality. In cases of normal distribution, paired *t*-tests were used to compare aortic growth between preoperative and 3-year measurements. In cases of non-normal distribution, a Wilcoxon rank sum test was used. A *P*-value of <0.05 was initially considered to be statistically significant. For all direct comparisons, Bonferroni’s adjustment was performed. Using the Bonferroni correction for 3 individual comparisons, a *P*-value of <0.0167 was considered significant in all individual comparisons.

## RESULTS

### Baseline demographics

A total of 47 patients were included in the original DARTS trial with 29 patients having 3-year follow-up imaging available at the time of data analysis. One patient was excluded from the original analysis as the AMDS was used off-label in the context of an iatrogenic aortic dissection at the time of the index procedure. Data were collected and compiled into the original dataset consisting of patients who underwent ATAD I repair with the AMDS from March 2017 to January 2019.

The mean age of the study population was 62.5 years and 31 (67.4%) patients were male. The prevalence of preoperative comorbidities in the study cohort included hypertension (58.7%), coronary artery disease (17.4%), chronic obstructive pulmonary disease (13.0%), diabetes (13.0%), previous stroke (13.0%) and chronic renal insufficiency (10.9%) (Table 2). Successful device deployment was achieved in all patients. Thirty-day mortality for the overall cohort was 13.0% and 1-year mortality was 19.6%. However, any patients who did not survive until their 3-year follow-up imaging were not included in this analysis, as this was a study of aortic remodelling at 3 years of follow-up.

**Table 2:** Baseline demographics of the study population

Baseline demographics	Study population (*n* = 46)
Average age	62.5
Male (%)	31 (67.4)
Body mass index (kg/m^2^)	29.1
Hypertension (%)	27 (58.7)
Current smoker (%)	15 (32.6)
Coronary artery disease (%)	8 (17.4)
Prior arrhythmia (%)	6 (13.0)
Chronic obstructive pulmonary disease (%)	6 (13.0)
Diabetes (%)	6 (13.0)
Previous stroke (%)	6 (13.0)
Chronic renal insufficiency (%)	5 (10.9)
Prior MI (%)	4 (8.7)
History of malignancy (%)	4 (8.7)
Preoperative aortic aneurysm (%)	4 (8.7)
Mean aneurysm size (mm)	51
Clinical or radiographic malperfusion	26 (56.5)
Prior DVT (%)	3 (6.5)
Congestive heart failure (%)	2 (4.3)

MI: myocardial infarction; DVT: deep vein thrombosis.

### Change in aortic diameter measurements at 3 years with supra-aortic vessel false lumen communications

**Figure 3:**
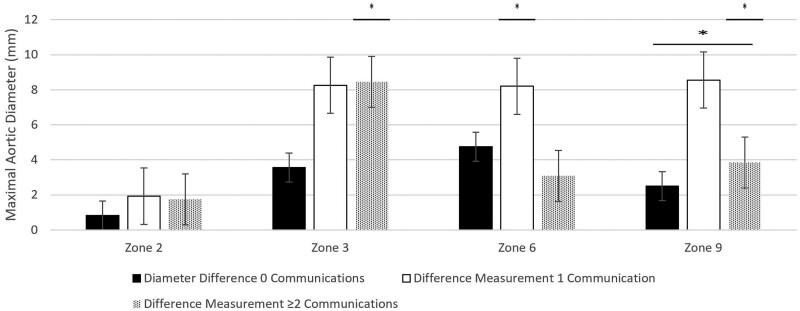
Changes in aortic diameter at 3 years with supra-aortic vessel false lumen communications.

Preoperative and postoperative data at zone 2 were available for 28 patients (7 with 0 communications, 8 with 1 communication and 13 with ≥2 communications). Measurements taken at zone 2 did not differ significantly between groups with 0, 1 or ≥2 supra-aortic branch vessel communications. The median aortic size preoperatively and postoperatively were 40.3 mm [interquartile range (IQR) 36.25–40.45 mm] and 36.9 mm (IQR 36.45–40.5 mm) for those with 0 head vessel FL communications (*P* = 0.61), 35.2 mm (IQR 34.4–37.65 mm) and 38.3 mm (IQR 36.85–40.15 mm) for those with 1 head vessel FL communication (*P* = 0.12) and 34.6 mm (IQR 32.5–35.8 mm) and 35.7 mm (IQR 33.7–38.9 mm) for those with ≥2 FL communications (*P* = 0.18) (Fig. 3). A one-way ANOVA did not identify significant differences between groups (*F*(2, 24) = [0.42], *P* = 0.66).

Preoperative and postoperative data at zone 3 were available for 28 patients (7 with 0 communications, 8 with 1 communication and 13 with ≥2 communications). For measurements taken at zone 3, patients with ≥2 FL communications had significant growth in contrast to patients with 0 or 1 FL communications. Aortic diameter changed from preoperative to postoperative measurements from 34.4 mm (IQR 32.7–35.9 mm) to 36.9 mm (IQR 34.45–43.15 mm) for patients with 0 FL communications (*P* = 0.11), 35.6 mm (IQR 32.7–36.63 mm) to 42.65 mm (IQR 38.13–44.33 mm) for patients with 1 FL communication (*P* = 0.02) and 32.6 mm (IQR 31.4–35.0 mm) to 40.9 mm (IQR 33.2–44.2 mm) for patients with ≥2 FL communications. The comparison from preoperative to postoperative measurements was non-normal and so a Wilcoxon rank sum test was performed and identified a significant difference between the groups (Fig. 3). A one-way ANOVA did not identify significant differences between groups (*F*(2, 24) = [1.25], *P* = 0.30).

Preoperative and postoperative data at zone 6 was available for 25 patients (7 with 0 communications, 7 with 1 communication and 11 with ≥2 communications). At zone 6, there was significant growth of the aorta for patients with 1 head vessel FL communication, while those with 0 or ≥2 FL communications did not. Aortic diameter changed from preoperative to postoperative measurements of a median 27.5 mm (IQR 25.95–29.95 mm) to 34.5 mm (IQR 29.9–36.1 mm) for patients with 0 FL communications (*P* = 0.02), 30.2 mm (IQR 29.2–32.7 mm) to 34.7 mm (IQR 34.2–43.7 mm) for patients with 1 FL communication (*P* = 0.008) and 27.1 mm (IQR 24.6–28.3 mm) to 29.5 mm (IQR 27.9–31.3 mm) for patients with ≥2 FL communications (*P* = 0.03) (Fig. 3). A one-way ANOVA did not identify significant differences between groups (*F*(2, 22) = [2.79], *P* = 0.08).

Preoperative and postoperative data at zone 9 were available for 24 patients (6 with 0 communications, 6 with 1 communication and 12 with ≥2 communications). Measurements taken at zone 9 identified significant growth only for patients with ≥2 FL communications at the head vessels. The median aortic diameter changed from preoperative to postoperative median measurements of 23.1 mm (IQR 21.3–23.8 mm) to 26.1 mm (IQR 23.4–28.6 mm) for patients with 0 FL communications (*P* = 0.13), 24.7 mm (IQR 23.0–24.8 mm) to 32.2 mm (IQR 27.7–32.9 mm) for patients with 1 FL communication (non-normal distribution, nonsignificant difference on Wilcoxon rank test) and 22.9 mm (IQR 20.7–23.6 mm) to 26.6 mm (IQR 24.6–28.3 mm) for patients with ≥2 FL communications (*P* = 0.002) (Fig. 3). A one-way ANOVA identified significant differences between groups (*F*(2, 20) = [3.83], *P* = 0.04).

### Change in aortic diameter at 3 years with visceral false lumen communications

**Figure 4:**
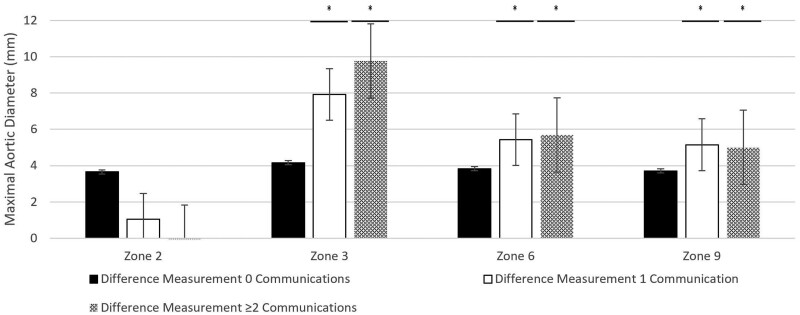
Changes in aortic diameter at 3 years with visceral vessel false lumen communications.

Preoperative and postoperative data at zone 2 was available for 28 patients (9 with 0 communications, 12 with 1 communication and 7 with ≥2 communications). There was no association between visceral FL communications and growth at zone 2. The median aortic diameter changed from preoperative to postoperative measurements of 35.1 mm (IQR 32.7–40.3 mm) to 40.1 mm (IQR 36.9–40.8 mm) for those with 0 FL communications (*P* = 0.027), 35.5 mm (IQR 34.6–38.7 mm) to 37.7 mm (IQR 25.6–39.3 mm) for those with 1 FL communication (*P* = 0.29) and 34.6 mm (IQR 33.0–36.3 mm) to 34.9 mm (IQR 34.0–35.5 mm) for those with ≥2 FL communications (*P* = 0.89) (Fig. 4). A one-way ANOVA did not identify significant differences between groups (*F*(2, 24) = [1.76], *P* = 0.19).

Preoperative and postoperative data at zone 3 was available for 28 patients (9 with 0 communications, 12 with 1 communication and 7 with ≥2 communications). For measurements taken at zone 3, there was significant growth in aortic diameter for patients with 1 and ≥2 FL communications. For patients with 0 FL communications, the median aortic diameter changed from preoperative to postoperative measurements of 32.6 mm (IQR 31.3–34.4 mm) to 36.8 mm (IQR 32.7–40.3 mm) (*P* = 0.031), 35.6 mm (IQR 33.8–36.5 mm) to 42.9 mm (IQR 38.8–46.2 mm) for those with 1 FL communication (*P* = 0.003) and 31.9 mm (IQR 31.0–34.3 mm) to 40.9 mm (IQR 36.2–42.7 mm) for those with ≥2 FL communications (non-normal distribution, significant difference on Wilcoxon rank test) (Fig. 4). A one-way ANOVA did not identify significant differences between groups (*F*(2, 24) = [1.05], *P* = 0.37).

Preoperative and postoperative data at zone 6 was available for 25 patients (8 with 0 communications, 11 with 1 communication and 6 with ≥2 communications). Measurements taken at zone 6 found nonsignificant growth for patients with 0 FL communications with preoperative to postoperative change in median aortic diameter of 27.5 mm (IQR 23.6–28.1 mm) (*P* = 0.06), while in contrast there was significant growth for patients with 1 FL communication of 30.0 mm (IQR 28.7–31.5 mm) to 34.0 mm (IQR 30.6–27.3 mm) (*P* = 0.009) and ≥2 FL communications of 26.2 mm (IQR 25.0–27.5 mm) to 32.4 mm (IQR 30.1–34.3 mm) (*P* = 0.0165) (Fig. 4). A one-way ANOVA did not identify significant differences between groups (*F*(2, 22) = [0.32], *P* = 0.73).

Preoperative and postoperative data at zone 9 was available for 24 patients (7 with 0 communications, 10 with 1 communication and 7 with ≥2 communications). Measurements taken at zone 9 identified nonsignificant growth for patients with 0 FL communications with median changes in aortic diameter from preoperative to postoperative measurements of 22.3 mm (IQR 20.6–24.3 mm) to 24.0 mm (IQR 20.6–26.5 mm) (non-normal distribution, no significant difference on Wilcoxon rank test) but significant growth for patients with 1 FL communication of 24.2 mm (IQR 22.8–24.8 mm) to 28.7 mm (IQR 27.5–31.5 mm) (*P* = 0.0003) and ≥2 FL communications of 22.5 mm (IQR 21.6–23.2 mm) to 26.2 mm (IQR 25.4–28.7) (*P* = 0.002) (Fig. 4). A one-way ANOVA did not identify significant differences between groups (*F*(2, 20) = [0.18], *P* = 0.84).

### Required reinterventions

A total of 7 patients from the original DARTS trial have required 9 aortic-related reinterventions to date (Table 3). Reintervention was required for progression of aortic dissection or aneurysm (6) or right ventricular dysfunction (1). Reinterventions included Bentall procedure (2), thoracic endovascular aortic repair (1) and other percutaneous vascular stenting or plugs (1 each). Among patients requiring supra-aortic vessel reinterventions, all patients had between 1 and 3 head vessel FL communications. Among patients requiring descending aortic or visceral branch interventions, patients had between 0 and 1 visceral FL communications (Table 3).

**Table 3:** Details of aortic-related reinterventions

Patient	Type of procedure	Procedure indication	Time between AMDS procedure and reintervention (days)	Intervention	Number of head vessel false lumen communications	Number of visceral false lumen communications
1	Percutaneous	Dissection Progression	2	Mesenteric stent	1	0
2	Percutaneous	Dissection Progression	81	Supra-aortic vessel stenting	2	3
3	Surgical	Dissection Progression	32	Bentall procedure	2	0
4	Percutaneous Percutaneous Percutaneous	New or worsening organ malperfusion Aortic aneurysm enlargement Aortic aneurysm enlargement	3 998 1304	Left renal stent Thoracic endovascular aortic repair Left subclavian artery plug	1	1
5	Surgical	Right ventricular dysfunction	5	CABG x3, Bentall procedure	1	3
6	Percutaneous	Dissection Progression	12	Carotid stent	3	1
7	Surgical	Dissection Progression	1188	Subclavian artery embolization with vascular plug	3	0

## DISCUSSION

**Figure 5:**
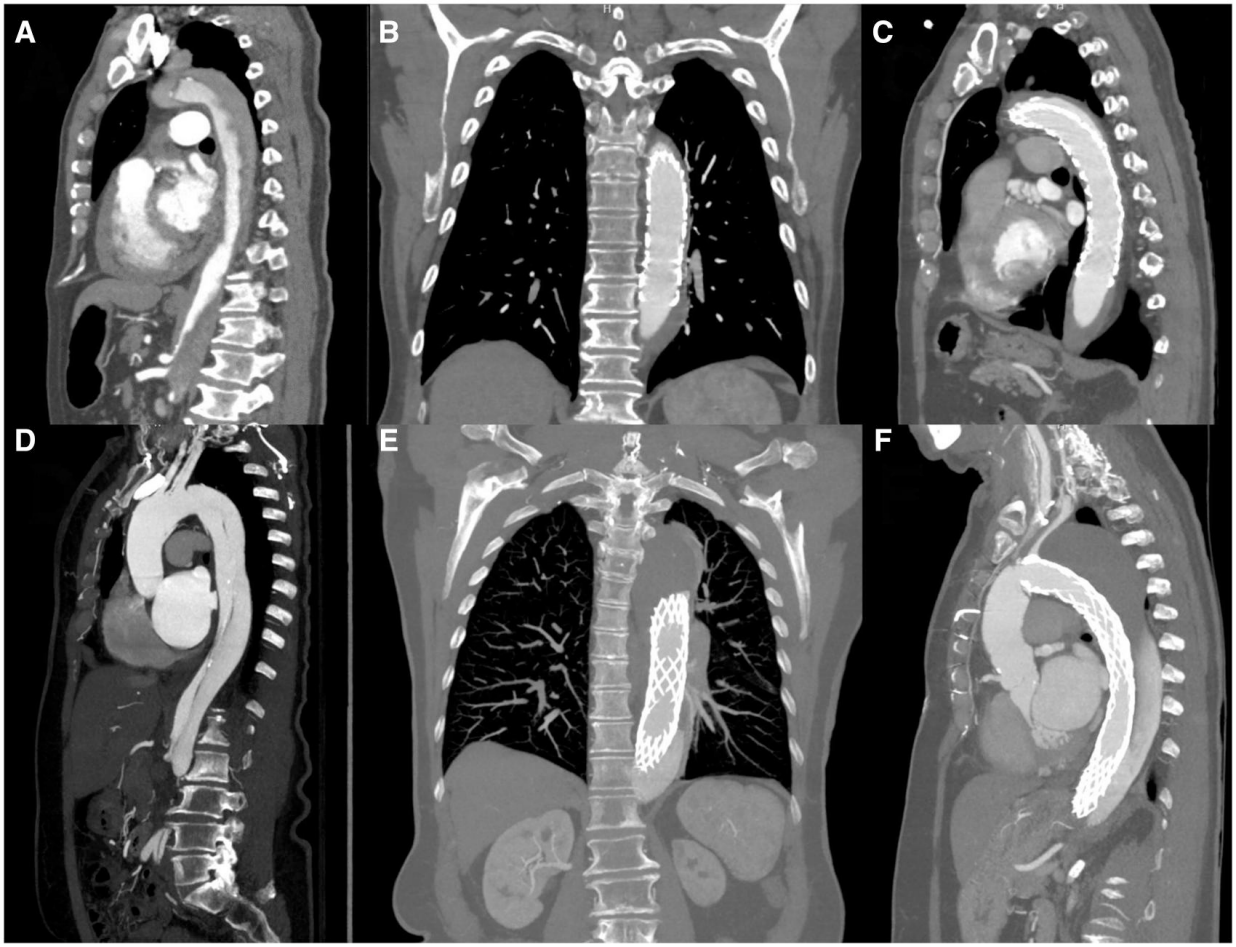
Preoperative and postoperative computerized tomography scans from a case of positive aortic remodelling following AMDS hybrid prosthesis implantation (**A**–**C**) and a case of adverse remodelling following AMDS hybrid prosthesis implantation (**D**–**F**).

The original DARTS study demonstrated the safety and efficacy of the AMDS [9–11]. While previous analyses of the DARTS trial data set showed positive aortic remodelling following AMDS implantation, some patients continued to have aortic growth although it is unclear which factors predict positive or negative remodelling (Fig. 5). This study found a correlation between the location of FL communications and aortic remodelling. First, in patients who underwent ATAD I repair with the AMDS, preoperative FL communications predicted postoperative local aortic growth. Patients with greater numbers of head vessel communications with the FL were more likely to have proximal growth in zone 3. Similarly, more FL communications at the visceral level predicted distal growth at zones 6 and 9 but did not show a clear association with proximal growth. Among the patients who required aortic-related reintervention, all patients with supra-aortic reinterventions had head vessel FL communications. The 2 patients requiring descending aortic or visceral vessel reinterventions had either 0 or 1 visceral FL communications.

This data demonstrates that preoperative FL communications may predict local growth of the aorta following ATAD I repair. Significant research has been dedicated in recent years to the identification of predictive factors for the remodelling of the aorta following aortic dissection repair, although a large proportion of this literature has focused on type B aortic dissections. Burris *et al.* [4] utilized four-dimensional magnetic resonance imaging (MRI) in patients with type B aortic dissections and found a correlation between FL ejection fraction and FL flow with aortic enlargement. Evangelista *et al.* followed 131 patients with type A or type B aortic dissection using CT and MRI to follow aortic remodelling. They found that aortic-related events including death or required subsequent endovascular intervention were predicted by factors such as aortic size and FL flow [13]. Van Bogerijen *et al.* conducted a review and meta-analysis of the literature investigating predictors of aortic growth in patients with uncomplicated type B aortic dissection. They identified numerous predictors of aortic growth of which anatomical features were prevalent including aortic size, FL diameter, patent or partially thrombosed FL, the shape of the aorta and features of entry tear(s) [12].

Notably, when discussing aortic remodelling, previous literature has largely studied patients with type B aortic dissections with a focus on entry tears and flow into the FL via these channels to predict aortic remodelling. There has been a paucity of literature investigating the impact of FL communications with head and visceral branch vessels resulting in FL flow and pressurization.

One study that did investigate the influence of FL side branches in patients with aortic dissection, was conducted by Rudenick *et al.* [18]. This group used MRI to evaluate patients with chronic descending aortic dissections following either surgical repair of ATAD I or medical management of type B dissection. Flow patterns through the aorta and FL including timing of flow and flow rate in mL/s were evaluated, including the influence of visceral branch vessels arising from the FL. They identified an increase in diastolic FL flow at the level of the diaphragm in patients with dominant branches arising from the FL with an associated decrease in systolic flow reversal. Patients with more FL side branches demonstrated flattening of the diastolic flow rate and reduced flow reversal after systole [18]. The data from this study highlight the importance of FL communications with visceral branch vessels and support the notion that these branches influence FL flow rate and presumably remodelling, although the relationship with long-term aortic remodelling was not identified in this study. These same principles are likely translatable to patients with acute type A dissections extending into the descending thoracic aorta, as is demonstrated in the data from the present study.

This study has demonstrated an association between branch vessel FL communications and local aortic remodelling following repair of ATAD I with the AMDS. While patients who required reintervention tended to have local FL communications, with only 7 patients requiring aortic-related reinterventions, statistical power is low and it is difficult to identify a definitive relationship with a relatively small sample size. With various approaches available to manage ATAD I and continued debate regarding the optimal management of these patients, predictors of positive or adverse remodelling may help to inform decision-making regarding the initial surgical repair of ATAD I. In the case of the AMDS hybrid prosthesis as with other medical devices, there are pros and cons to its use. It helps to facilitate aortic remodelling, is beneficial for use in patients with malperfusion and does not require additional skill or operative times, while it is not indicated in those without a distal landing zone and requires careful consideration in patients with distal entry tears where its use may not be optimal. Until now, predictors of aortic remodelling following AMDS hybrid prosthesis implantation were not known. The identification of FL communication patterns that may predict postoperative aortic remodelling may help to guide initial interventions by allowing for the selection of a repair approach that will result in the most favourable outcome for each patient. Although a total arch repair may increase the procedural risk compared to a standard hemiarch repair, in patients with an increased risk of adverse arch remodelling a total arch repair would address this issue, precluding FL perfusion by head vessel branches proximal to the distal suture line. While the findings of this study demonstrate an association between branch vessel FL communications and aortic remodelling, given the limited sample sizes currently available, further investigation is warranted to confirm this relationship. Additional analyses with larger sample sizes and various surgical repairs should be completed to improve the understanding of the impact of FL communications on aortic remodelling following aortic dissection repair and to identify statistically significant differences that this study may not have been powered to detect.

### Limitations

The main limitation of this study is the limited sample size. While the original DARTS trial was the first clinical trial with a sizeable cohort to target patients with ATAD I, the sample size remains relatively small and may impede the detection of clear relationships. Furthermore, there were no control patients included in this study as the DARTS trial was a single-arm trial. As only patients who received AMDS repair were included, the results of this study may not be generalizable to patients who received other surgical repairs for ATAD I. Not all patients were included in all measurements due to the AMDS hybrid prosthesis limiting views, a bovine arch, the CT scan being limited to the chest, imaging artefact, imaging without contrast, poor overall image quality or separation of the image series, although this is difficult to definitively determine on a case-by-case basis given the trial data has been anonymized. Finally, preoperative variables were not adjusted for between groups introducing potential confounding variables.

## CONCLUSIONS

Aortic remodelling following ATAD I and repair using the AMDS may be predicted by local FL communications with branch vessels. In this study, patients undergoing ATAD I repair with the AMDS were more likely to experience significant aortic growth in zone 3 with a greater number of head vessel FL communications. Similarly, patients with a greater number of FL communications with visceral vessels had a greater degree of aortic growth in zones 3, 6 and 9 compared to patients with fewer FL communications. Predictors of future aortic remodelling following ATAD I may help to guide initial surgical management, although future studies utilizing larger sample sizes and various repair types will be required to confirm this relationship and to identify optimal surgical approaches to patients with various anatomy following ATAD I.

## Data Availability

Data associated with the article is available on reasonable request to the corresponding author. **Author contributions: Ryaan EL-Andari:** Conceptualization; Formal analysis; Investigation; Writing—original draft. **Sabin J. Bozso:** Conceptualization; Formal analysis; Investigation; Writing—original draft. **Jennifer Chung:** Conceptualization; Investigation; Writing—review & editing. **Maral Ouzounian:** Conceptualization; Investigation; Writing—review & editing. **Jeevan Nagendran:** Conceptualization; Investigation; Writing—review & editing. **Michael C. Moon:** Conceptualization; Investigation; Supervision; Writing—review & editing. **Reviewer information:** European Journal of Cardio-Thoracic Surgery thanks Gabriele Piffaretti, David C. Reineke, Sven Martens, Daniel-Sebastian Dohle for their contribution to the peer review process of this article.

## Notes

Presented at the EACTS Annual Meeting 2023, Vienna, Austria.

## ABBREVIATIONS

ANOVA: Analysis of variance
ATAD I: Acute DeBakey type I aortic dissection
CT: Computerized tomography
DARTS: Dissected Aorta Repair Through Stent
FL: False lumen
IQR: Interquartile range
MRI: Magnetic resonance imaging
